# Measuring Uncertainty in the Negation Evidence for Multi-Source Information Fusion

**DOI:** 10.3390/e24111596

**Published:** 2022-11-02

**Authors:** Yongchuan Tang, Yong Chen, Deyun Zhou

**Affiliations:** 1 School of Microelectronics, Northwestern Polytechnical University, Xi’an 710072, China; 2 School of Big Data and Software Engineering, Chongqing University, Chongqing 401331, China

**Keywords:** Dempster–Shafer evidence theory, uncertainty measure, negation evidence, belief entropy, multi-source information fusion

## Abstract

Dempster–Shafer evidence theory is widely used in modeling and reasoning uncertain information in real applications. Recently, a new perspective of modeling uncertain information with the negation of evidence was proposed and has attracted a lot of attention. Both the basic probability assignment (BPA) and the negation of BPA in the evidence theory framework can model and reason uncertain information. However, how to address the uncertainty in the negation information modeled as the negation of BPA is still an open issue. Inspired by the uncertainty measures in Dempster–Shafer evidence theory, a method of measuring the uncertainty in the negation evidence is proposed. The belief entropy named Deng entropy, which has attracted a lot of attention among researchers, is adopted and improved for measuring the uncertainty of negation evidence. The proposed measure is defined based on the negation function of BPA and can quantify the uncertainty of the negation evidence. In addition, an improved method of multi-source information fusion considering uncertainty quantification in the negation evidence with the new measure is proposed. Experimental results on a numerical example and a fault diagnosis problem verify the rationality and effectiveness of the proposed method in measuring and fusing uncertain information.

## 1. Introduction

Uncertain information exists widely in practical applications [1,2]. One of the effective methods for uncertain information modeling, reasoning and fusion is Dempster–Shafer evidence theory [3,4]. It has been widely used in real applications, such as pattern recognition [5], classification [6,7,8], clustering [9,10,11], decision making under uncertainty [12,13,14,15], risk analysis in complicated systems with knowledge [16,17], human reliability analysis considering dependence information [18,19], supplier selection [20], failure mode and effects analysis in [21,22] and so on [23,24]. Although Dempster–Shafer evidence theory has some advantages and is widely used in uncertain information modeling and reasoning, it has some open issues in the theory itself. First, the classical Dempster’s rule of combination cannot be directly used for high conflict evidence fusion because it may cause counter-intuitive fusion results [25,26,27]. Second, the modeling of uncertain information with the basic probability assignment (BPA) is still mainly based on empirical methods [28,29]. Third, how to measure the BPA uncertainty degree still requires further research [30,31]. In this paper, we extend the uncertainty measure for a BPA to the the negation of a BPA and propose an improved multi-source information fusion method.

Shannon entropy is a well-known theory for uncertainty measure in the probabilistic framework, which has attracted much attention in real applications [32,33,34]. However, due to the reason that a mass function is the generalized probability assigned on the power set of the frame of discernment (FOD), Shannon entropy cannot be used directly among mass functions in the framework of Dempster–Shafer evidence theory. To address this open issue, many uncertainty measures in the Dempster–Shafer framework are defined, e.g., the conflict measure [35], the measure of interval belief functions [36], the Deng entropy [37], the soft likelihood function [38], the correlation coefficient [39], the fractal-based belief entropy [40] and so on [30]. The uncertainty quantification of evidence itself is still an open issue [31]. Among these uncertainty measures, the Deng entropy is widely used in many applications [41]. This work will adopt the Deng entropy to measure the uncertainty in the negation evidence.

The negation of BPA is used for modeling the uncertain information in the frame of Dempster–Shafer evidence theory [42,43,44]. How to measure the uncertainty for the negation of BPA is helpful for uncertain information management and should be addressed. Based on Deng entropy and the negation of mass function, this paper proposes an improved belief entropy of negation BPA to handle the uncertain measure of BPA. The improved belief entropy of negation BPA provides a novel view to measure the uncertainty of BPA. After that, an improved data fusion method considering the belief entropy of negation evidence is presented. In the presented method, the improved belief entropy of negation evidence is used to pre-process the conflict data by measuring the BPA uncertainty degree in each body of evidence (BOE). Then, the final weight for each BOE is presented based on the measure results. Finally, the multi-source data is fused by Dempster’s rule of combination. Several numerical examples are illustrated to analyze the performance of the improve belief entropy of negation evidence. Moreover, the new data fusion method is applied on an application of fault diagnosis to test and verify the effectiveness of the new method.

The rest of this work is organized as follows. Section 2 is the preliminaries on Dempster–Shafer evidence theory, the negation evidence, and the belief entropy. In Section 3, the improved belief entropy for negation evidence is proposed as well as a new approach of multi-source information fusion using the improved belief entropy for uncertainty quantification. Experiments of the proposed method on artificial data and a case study in fault diagnosis are given in Section 4. Section 5 is the conclusion.

## 2. Preliminaries

### 2.1. Dempster–Shafer Evidence Theory

Define that $\Omega =\left\{\theta_{1},\theta_{2},\ldots ,\theta_{i},\ldots ,\theta_{N}\right\}$ is a finite nonempty set with *N* mutually exclusive and exhaustive events, and Ω is called the frame of discernment (FOD). The power set of Ω denoted as $2^{\Omega }$ has $2^{N}$ elements [3,4]:

(1) $$2^{\Omega }=\left\{\emptyset ,\left\{\theta_{1}\right\},\left\{\theta_{2}\right\},\ldots ,\left\{\theta_{N}\right\},\left\{\theta_{1},\theta_{2}\right\},\ldots ,\left\{\theta_{1},\theta_{2},\ldots ,\theta_{i}\right\},\ldots ,\Omega \right\}.$$

A mass function *m*, also named the basic belief assignment (BBA) or basic probability assignment (BPA), is defined as a mapping function from the power set of FOD $2^{\Omega }$ to the interval [0, 1]. It satisfies [3,4]:

(2) $$m\left(\emptyset \right)=0,\sum_{A\in \Omega }m\left(A\right)=1.$$

If $m\left(A\right)>0$, then *A* is defined as a focal element.

A body of evidence (BOE) means the focal sets and the associated belief value that can be denoted as follows:

(3) $$\left(ℜ,m\right)=\left\{\langle A,m\left(A\right)\rangle :A\in 2^{\Omega },m\left(A\right)>0\right\}.$$

where *ℜ* is a subset of the power set $2^{\Omega }$. For each A∈ℜ, there is a nonzero BPA value $m\left(A\right)$.

Two independent mass functions $m_{1}$ and $m_{2}$ can be combined with Dempster’s rule of combination defined as [3,4]:

(4) $$m\left(A\right)=\left(m_{1}⊕m_{2}\right)\left(A\right)\;=\frac{1}{1-k}\sum_{B\cap C=A}m_{1}\left(B\right)m_{2}\left(C\right),$$

where *k* can be regarded as a normalization factor defined as [3,4]:

(5) $$k=\sum_{B\cap C=\emptyset }m_{1}\left(B\right)m_{2}\left(C\right).$$

### 2.2. The Negation Evidence

The negation information represents a kind of uncertain information in probability framework [42]. In Dempster–Shafer evidence theory, Yin et al. [43] proposed a new method to calculate the negation of the BPA for uncertain information modeling and processing.

In the BOE, $e_{i}$ means the ith focal element. For each focal element $e_{i}$, $m(e_{i})$ is the belief value of the ith focal element, and the negation of $m(e_{i})$ is denoted as $\bar{m}$$\left(e_{i}\right)$. The general formula of the negation evidence can be derived as [43]:

(6) $$\bar{m}\left(e_{i}\right)=\frac{1-m(e_{i})}{n-1},$$

where *n* is the number of focal elements in the BOE. It is obvious that if the belief value is one for only one focal element, there is no uncertain information in this case and it is a certain event.

### 2.3. Belief Entropy

Belief entropy is proposed for the uncertainty measure in the framework of Dempster–Shafer evidence theory. As a belief entropy, Deng entropy [37] is regarded as a generalization of Shannon entropy in the Dempster–Shafer framework, and it has been widely used in applications [41]. Deng entropy, denoted as $E_{d}$, is defined as follows [37]:

(7) $$E_{d}\left(m\right)=-\sum_{A\subseteq X}m\left(A\right)\log_{2}\frac{m\left(A\right)}{2^{\left|A\right|}-1},$$

where $\left|A\right|$ denotes the cardinality of the proposition *A*, and *X* is the FOD.

## 3. An Improved Multi-Source Information Fusion Method Based on Measuring the Uncertainty of Negation Evidence

### 3.1. Improved Belief Entropy of Negation Evidence

Base on the belief entropy of belief functions in Equation (7), an improved belief entropy of negation evidence is defined as follows:

(8) $$E_{n}\left(m\right)=-\sum_{A\subseteq X}\bar{m}\left(A\right)\log_{2}\frac{\bar{m}\left(A\right)}{2^{\left|A\right|}-1},$$

where *A* is the focal element of BOE, $\left|A\right|$ is the cardinality of *A*, and $\bar{m}$ is the negation evidence of the mass function *m*; $\bar{m}$ can be calculated as follows based on the negation evidence defined in Equation (6):

(9) $$\bar{m}\left(A\right)=\frac{1-m(A)}{n-1},$$

where *n* is the number of focal elements in the BOE.

The belief entropy of negation evidence addresses the uncertainty measure of the negation of BPA. Two numerical examples are given to explain how to calculate the belief entropy of negation evidence.

Example 1: Assume the FOD is {a, b, c}. The mass functions are m(a)=0.2, m(b)=0 and m(c)=0.1. The belief entropy of negation evidence can be calculated with the following steps. First, calculating the negation of mass functions:

$$\bar{m}\left(a\right)=\frac{1-m(a)}{n-1}=0.4,\bar{m}\left(b\right)=\frac{1-m(b)}{n-1}=0.15,\bar{m}\left(c\right)=\frac{1-m(c)}{n-1}=0.45.$$

Then, the belief entropy of negation evidence can be calculated as follows:

$$E_{n}\left(m\right)=-0.4\times \log_{2}\frac{0.4}{2^{1}-1}-0.15\times \log_{2}\frac{0.15}{2^{1}-1}-0.45\times \log_{2}\frac{0.45}{2^{1}-1}=1.4577.$$

Example 2: Assume the FOD is {a, b}. The mass functions are m(a)=0.1, m(b)=0.4 and m(a,b)=0.5. The belief entropy of negation evidence can be calculated with the following steps. First, calculating the negation of mass functions:

$$\bar{m}\left(a\right)=\frac{1-m(a)}{n-1}=0.45,\bar{m}\left(b\right)=\frac{1-m(b)}{n-1}=0.3,\bar{m}\left(a,b\right)=\frac{1-m(a,b)}{n-1}=0.25.$$

Then, the belief entropy of negation evidence can be calculated as follows:

$$E_{n}\left(m\right)=-0.45\times \log_{2}\frac{0.45}{2^{1}-1}-0.3\times \log_{2}\frac{0.3}{2^{1}-1}-0.25\times \log_{2}\frac{0.25}{2^{2}-1}=1.5395.$$

### 3.2. Multi-Source Information Fusion Considering the Uncertainty of Negation Evidence

**Figure 1:**
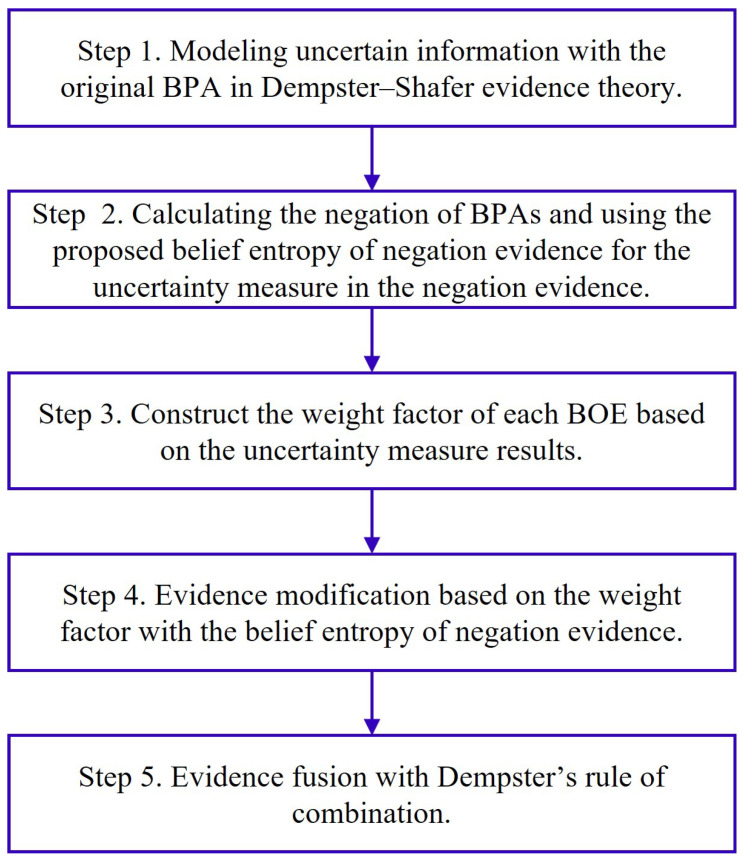
The flowchart of multi-source information fusion based on the belief entropy of negation evidence.

A new method of multi-source information fusion using the improved belief entropy of negation evidence is proposed in this section, as shown in Figure 1. The steps are presented as follows.

Step 1 Modeling uncertain information with the original BPA in Dempster–Shafer evidence theory.
  In real applications, due to the diversity styles of information, many methods for generation of BPAs are proposed [28,45]. Currently, a method of generating BPA automatically for different sceneries in practical applications is not available and there are many choices.
Step 2 Calculating the negation of BPAs and using the proposed belief entropy of negation evidence for the uncertainty measure in the negation evidence.
  For the *i*th BOE (i=1,2,⋯), the corresponding uncertain degree with the belief entropy of negation evidence $E_{n}$ is calculated as follows:
  (10) $$E_{n}\left(m_{i}\right)=-\sum_{A\subseteq X}{\bar{m}}_{i}\left(A\right)\log_{2}\frac{{\bar{m}}_{i}\left(A\right)}{2^{\left|A\right|}-1},$$
  (11) $${\bar{m}}_{i}\left(A\right)=\frac{1-m_{i}\left(A\right)}{n-1}.$$
Step 3 Construct the weight factor of each BOE based on the uncertainty measure results.
  There may be conflict among different sources of evidence. The weight factor is based on the uncertainty measure and for balancing different information sources. The relative weight factor for the *i*th BOE (i=1,2,⋯,m) among all the available number of BOEs, denoted as $w_{i}$, is defined as follows:
  (12) $$w_{i}=\frac{E_{n}\left(m_{i}\right)}{\sum_{i=1}^{m}E_{n}\left(m_{i}\right)}.$$
Step 4 Evidence modification based on the weight factor with the belief entropy of negation evidence.
  Based on the weight factor of each BOE, the weighted mass function of each proposition is calculated for final data fusion. For each proposition *A* in the BOE, the weighted mass function can be calculated as follows:
  (13) $$m_{w}\left(A\right)=\sum_{i=1}^{n}w_{i}m_{i}\left(A\right).$$
Step 5 Evidence fusion with Dempster’s rule of combination.
  The BPAs of multi-source information have been measured and modified based on the proposed measure and now are ready for information fusion with Dempster’s rule of combination. For each proposition *A* in the BOE, the combination result of modified evidence can be calculated by calculating Dempster’s rule of combination with (m−1) (m≥2) times:
  (14) $$m\left(A\right)={\left({\left({\left({\left(m_{w}⊕m_{w}\right)}_{1}⊕m_{w}\right)}_{2}...⊕m_{w}\right)}_{\left(m-2\right)}⊕m_{w}\right)}_{\left(m-1\right)}\left(A\right).$$

## 4. Experiment and Discussion

### 4.1. Experiment with Artificial Data

An experiment with artificial data in [27,46] is adopted to demonstrate the effectiveness and rationality of the proposed multi-source information fusion method. It is assumed that there are five independent information sources. Each information source can be modeled as an independent body of evidence. Since the traditional Dempster combination rule can be directly used for evidence fusion if there is no conflict, for the artificial data in [46] it is assumed that there is conflict evidence and the conflict is caused by an unreliable sensor or other unknown reasons. Thus, similar to [27], the artificial data with unreliable evidence in [46] is adopted to verify the effectiveness of the proposed method for multi-source information fusion with conflict data.

The artificial data in [27,46] are as follows. Consider a target recognition problem. Three potential targets are, respectively, denoted as *A*, *B* and *C* in the FOD denoted as X={A,B,C}. As presented in Table 1, the multi-source information is modeled as BPAs denoted as $m_{1}$, $m_{2}$, $m_{3}$, $m_{4}$ and $m_{5}$. Intuitively, the evidence from the second information source ($m_{2}$) is contrary to the other four pieces of evidence, and *A* will be the recognized target according to the highest belief value on *A* in the other four sources of information.

The steps of the proposed method of multi-source information fusion considering the uncertainty of negation evidence in the experiment are as follows.

For the first step, the BPAs are adopted from [27,46], and the result is shown in Table 1.

For the second step, with Equations (10) and (11), the belief entropy of negation evidence of $m_{1}$ is calculated as follows:

$${\bar{m}}_{1}\left(A\right)=\frac{1-m_{1}\left(A\right)}{n-1}=0.295,$$

$${\bar{m}}_{1}\left(B\right)=\frac{1-m_{1}\left(B\right)}{n-1}=0.355,$$

$${\bar{m}}_{1}\left(C\right)=\frac{1-m_{1}\left(C\right)}{n-1}=0.35,$$

$$E_{n}\left(m_{1}\right)=-\sum_{A\subseteq X}\frac{\left|A\right|{\bar{m}}_{1}\left(A\right)}{\left|X\right|}\log_{2}\frac{{\bar{m}}_{1}\left(A\right)}{2^{\left|A\right|}-1}=1.5801.$$

Similarly, the belief entropy of negation evidence of $m_{2}$ to $m_{5}$ can be calculated, and the results are as follows: $E_{n}\left(m_{2}\right)=0.7345$, $E_{n}\left(m_{3}\right)=2.0286$, $E_{n}\left(m_{4}\right)=2.0447$ and $E_{n}\left(m_{5}\right)=2.06762$.

For the third step, with Equation (12), the weight of each body of evidence is calculated as follows:


$$\begin{array}{c} w_{1}=\frac{E_{n}\left(m_{1}\right)}{\sum_{i=1}^{5}E_{n}\left(m_{i}\right)}=0.1869,w_{2}=\frac{E_{n}\left(m_{2}\right)}{\sum_{i=1}^{5}E_{n}\left(m_{i}\right)}=0.0869,w_{3}=\frac{E_{n}\left(m_{3}\right)}{\sum_{i=1}^{5}E_{n}\left(m_{i}\right)}=0.2399,\\ w_{4}=\frac{E_{n}\left(m_{4}\right)}{\sum_{i=1}^{5}E_{n}\left(m_{i}\right)}=0.2418,w_{5}=\frac{E_{n}\left(m_{5}\right)}{\sum_{i=1}^{5}E_{n}\left(m_{i}\right)}=0.2445. \end{array}$$

For the fourth step, the mass function of each proposition after modification in Table 1 can be calculated with Equation (13). The calculation results are as follows:

$$m_{w}\left(A\right)=\sum_{i=1}^{5}w_{i}m_{i}\left(A\right)=0.4955,m_{w}\left(B\right)=\sum_{i=1}^{5}w_{i}m_{i}\left(B\right)=0.1978,$$

$$m_{w}\left(C\right)=\sum_{i=1}^{5}w_{i}m_{i}\left(C\right)=0.0647,m_{w}\left(A,C\right)=\sum_{i=1}^{5}w_{i}m_{i}\left(A,C\right)=0.2420.$$

Finally, for the fifth step, there are five original pieces of evidence; thus, with Dempster’s rule of combination and Equation (14), the modified evidence is fused four times. The information fusion process and results are listed as follows:

$$m\left(A\right)={\left({\left({\left({\left(m_{w}⊕m_{w}\right)}_{1}⊕m_{w}\right)}_{2}⊕m_{w}\right)}_{3}⊕m_{w}\right)}_{4}\left(A\right)=0.9863,$$

$$m\left(B\right)={\left({\left({\left({\left(m_{w}⊕m_{w}\right)}_{1}⊕m_{w}\right)}_{2}⊕m_{w}\right)}_{3}⊕m_{w}\right)}_{4}\left(B\right)=0.0013,$$

$$m\left(C\right)={\left({\left({\left({\left(m_{w}⊕m_{w}\right)}_{1}⊕m_{w}\right)}_{2}⊕m_{w}\right)}_{3}⊕m_{w}\right)}_{4}\left(C\right)=0.0086,$$

$$m\left(A,C\right)={\left({\left({\left({\left(m_{w}⊕m_{w}\right)}_{1}⊕m_{w}\right)}_{2}⊕m_{w}\right)}_{3}⊕m_{w}\right)}_{4}\left(A,C\right)=0.0038.$$

The multi-source information fusion results with the proposed method and other methods in the experiment are presented in Table 2. With the proposed method, it can be inferred that target *A* is the recognized target, which is consistent with other methods in [27,46,47,48]. Compared to the fusion results based on the other methods in [46,47], the proposed method has the highest belief (98.63%) on the recognized target *A*. By considering the uncertainty of negation evidence, the proposed method contributes to a higher belief degree on the expected target than the methods in [46,47]. The methods in [27,48] have higher belief degree on target *A* than the proposed method. However, it should be noted that both Yuan et al.’s method in [48] and Xiao’s method in [27] use the distance-based measure and the belief entropy-based measure simultaneously in the flowchart of the method. Since both the distance-based measure and the belief entropy-based measure are based on BPA functions, there may exist coupling weight factors. Thus, these two methods should be used cautiously in some cases.

### 4.2. Experiment in Fault Diagnosis

In this section, the proposed method is applied to an application in fault diagnosis of a motor rotor. The practical data in [49] is adopted for the experiment. Suppose that there are three types of faults in a motor rotor. F1 means {*Rotor unbalance*}, F2 means {*Rotor misalignment*} and F3 means {*Pedestal looseness*}. The vibration signals are collected from three vibration acceleration sensors which are placed in different installation positions. The acceleration vibration frequency amplitudes at the frequencies of *Freq*1, *Freq*2 and *Freq*3 are taken as the fault feature variables. The data for fault diagnosis modeled as BPAs are provided in Table 3: $m_{s_{1}}\left(\cdot \right)$, $m_{s_{2}}\left(\cdot \right)$ and $m_{s_{3}}\left(\cdot \right)$, respectively, denote the BOEs reported from these three vibration acceleration sensors.

Apply the proposed method for multi-source information fusion in fault diagnosis.

**Step 1**  Modeling uncertain information with BPA in Dempster–Shafer evidence theory.

In this paper, BPAs of sensor reports are directly adopted from [49], as represented in Table 3. For more details about generating BPAs of Table 3, please refer to [49].

**Step 2**  Using the proposed belief entropy of negation evidence for uncertainty measure of the negation of BPAs.

In the proposed method, the uncertainty of sensor reports is measured based on the belief entropy of negation evidence. With Equations (10) and (11), the negation evidence and the belief entropy of negation evidence of each BOE under the vibration acceleration frequency of *Freq*1 is calculated as follows:

$$\begin{array}{c} {\bar{m}}_{s1}\left(\left\{F2\right\}\right)=\frac{1-m_{s1}\left(\left\{F2\right\}\right)}{n-1}=0.0608,{\bar{m}}_{s1}\left(\left\{F3\right\}\right)=\frac{1-m_{s1}\left(\left\{F3\right\}\right)}{n-1}=0.3332,\\ {\bar{m}}_{s1}\left(\left\{F1,F2\right\}\right)=\frac{1-m_{s1}\left(\left\{F1,F2\right\}\right)}{n-1}=0.2816,{\bar{m}}_{s1}\left(\left\{F1,F2,F3\right\}\right)=\frac{1-m_{s1}\left(\left\{F1,F2,F3\right\}\right)}{n-1}=0.3244, \end{array}$$

$$E_{n}\left(m_{s_{1}}\right)=-\sum_{A\subseteq X}{\bar{m}}_{s_{1}}\left(A\right)\log_{2}\frac{{\bar{m}}_{s_{1}}\left(A\right)}{2^{\left|A\right|}-1}=3.1726.$$

$$\begin{array}{c} {\bar{m}}_{s2}\left(\left\{F2\right\}\right)=\frac{1-m_{s2}\left(\left\{F2\right\}\right)}{n-1}=0.1447,{\bar{m}}_{s2}\left(\left\{F3\right\}\right)=\frac{1-m_{s2}\left(\left\{F3\right\}\right)}{n-1}=0.3330,\\ {\bar{m}}_{s2}\left(\left\{F1,F2\right\}\right)=\frac{1-m_{s2}\left(\left\{F1,F2\right\}\right)}{n-1}=0.3118,{\bar{m}}_{s2}\left(\left\{F1,F2,F3\right\}\right)=\frac{1-m_{s2}\left(\left\{F1,F2,F3\right\}\right)}{n-1}=0.2104, \end{array}$$

$$E_{n}\left(m_{s_{2}}\right)=-\sum_{A\subseteq X}{\bar{m}}_{s_{1}}\left(A\right)\log_{2}\frac{{\bar{m}}_{s_{2}}\left(A\right)}{2^{\left|A\right|}-1}=3.0142.$$

$$\begin{array}{c} {\bar{m}}_{s3}\left(\left\{F2\right\}\right)=\frac{1-m_{s3}\left(\left\{F2\right\}\right)}{n-1}=0.2532,{\bar{m}}_{s3}\left(\left\{F3\right\}\right)=\frac{1-m_{s3}\left(\left\{F3\right\}\right)}{n-1}=0.3332,\\ {\bar{m}}_{s3}\left(\left\{F1,F2\right\}\right)=\frac{1-m_{s3}\left(\left\{F1,F2\right\}\right)}{n-1}=0.3286,{\bar{m}}_{s3}\left(\left\{F1,F2,F3\right\}\right)=\frac{1-m_{s3}\left(\left\{F1,F2,F3\right\}\right)}{n-1}=0.0849, \end{array}$$

$$E_{n}\left(m_{s_{3}}\right)=-\sum_{A\subseteq X}{\bar{m}}_{s_{1}}\left(A\right)\log_{3}\frac{{\bar{m}}_{s_{1}}\left(A\right)}{2^{\left|A\right|}-1}=2.6191.$$

Similarly, the belief entropy of negation evidence of sensor reports under *Freq*2 and *Freq*3 can be calculated with the proposed method. The measuring results with the proposed method are listed in Table 4.

**Step 3**  Construct the weight factor of each BOE based on the uncertainty measure result.

With Equation (12), for the vibration acceleration frequency of Freq1, the weight of each BOE for evidence modification is calculated as follows:

$$w_{S_{1}}=\frac{E_{n}\left(m_{s_{1}}\right)}{\sum_{i=1}^{3}E_{n}\left(m_{s_{i}}\right)}=\frac{3.1726}{3.1726+3.0142+2.6191}=0.3603,$$

$$w_{S_{2}}=\frac{E_{n}\left(m_{s_{2}}\right)}{\sum_{i=1}^{3}E_{n}\left(m_{s_{i}}\right)}=\frac{3.0142}{3.1726+3.0142+2.6191}=0.3423,$$

$$w_{S_{3}}=\frac{E_{n}\left(m_{s_{3}}\right)}{\sum_{i=1}^{3}E_{n}\left(m_{s_{i}}\right)}=\frac{2.6191}{3.1726+3.0142+2.6191}=0.2974.$$

The weight of different sensor reports under Freq2 and Freq3 is shown in Table 5.

**Step 4**  Evidence modification based on the weight factor with the belief entropy of negation evidence.

With Equation (13), the modified mass function for each information source on fault types with respect to Freq1 can be calculated as follows:

$$m_{w}\left(\left\{F2\right\}\right)=\sum_{i=1}^{3}w_{si}m_{i}\left(\left\{F2\right\}\right)=0.5597,$$

$$m_{w}\left(\left\{F3\right\}\right)=\sum_{i=1}^{3}w_{si}m_{i}\left(\left\{F3\right\}\right)=0.0005,$$

$$m_{w}\left(\left\{F1,F2\right\}\right)=\sum_{i=1}^{3}w_{si}m_{i}\left(\left\{F1,F2\right\}\right)=0.0823,$$

$$m_{w}\left(\left\{F1,F2,F3\right\}\right)=\sum_{i=1}^{3}w_{si}m_{i}\left(\left\{F1,F2,F3\right\}\right)=0.3575.$$

The modified mass function for Freq2 and Freq3 can also be calculated with Equation (13). The result is shown in Table 6.

**Step 5**  Evidence fusion with Dempster’s rule of combination.

With Equation (14), for the vibration acceleration frequency of Freq1, there are three information sources, and the modified mass function will be fused with Dempster’s rule of combination by two times, shown as follows:

$$m\left(\left\{F2\right\}\right)={\left({\left(m_{w}⊕m_{w}\right)}_{1}⊕m_{w}\right)}_{2}\left(\left\{F2\right\}\right)=0.9146,$$

$$m\left(\left\{F3\right\}\right)={\left({\left(m_{w}⊕m_{w}\right)}_{1}⊕m_{w}\right)}_{2}\left(\left\{F3\right\}\right)=0.0002,$$

$$m\left(\left\{F1,F2\right\}\right)={\left({\left(m_{w}⊕m_{w}\right)}_{1}⊕m_{w}\right)}_{2}\left(\left\{F1,F2\right\}\right)=0.0394,$$

$$m\left(\left\{F1,F2,F3\right\}\right)={\left({\left(m_{w}⊕m_{w}\right)}_{1}⊕m_{w}\right)}_{2}\left(\left\{F1,F2,F3\right\}\right)=0.0458.$$

The fusion results for Freq2 and Freq3 are shown in Table 7.

According to the fusion results in Table 7, the diagnosis result after evidence fusion is that F2 is the recognized target. The conflict of sensor reports in the application is overcome with the proposed method, even though the belief degree on F2 under Freq1 is 0.8176, 0.5658 and 0.2403, respectively. Table 8 is a comparison between the proposed methods and some other works. It shows that the diagnosis result of the proposed method is in accordance with the methods in [49,50], and the fault type is F2. Moreover, the proposed method has a higher belief degree on fault type F2 than the method in the literature under different frequencies.

A few reasons contribute to the effectiveness of the proposed conflict evidence fusion method. Firstly, the sensor data is preprocessed properly before applying the combination rules, which is a very important step in combining conflict evidence. Secondly, the new method is based on an improved belief entropy of negation evidence. The new measure can measure the negation uncertainty information in the Dempster–Shafer evidence theory framework, which contributes to a more accurate experiment result. Finally, the advantages in Dempster’s combination rule, such as satisfying the commutativity and associativity, guarantee the rationality of the fusion result.

### 4.3. Discussion and Limitation

A new method of measuring uncertainty in the negation evidence and its application in multi-source information fusion is proposed in this work. The contribution of the work can be summarized as follows. (1) An improved belief entropy is proposed to measure the uncertainty of negation evidence. Currently, all the measures in the framework of Dempster–Shafer evidence theory are designed for the original evidence, and there is no uncertainty measure for the negation evidence [31,41]. The improved belief entropy of the negation BPA function provides a new perspective to quantify the uncertainty degree of uncertain information. (2) An improved multi-source information fusion method considering the uncertainty in the negation information is proposed. Both the original evidence and the negation evidence can model uncertain information [42,44]. The negation evidence should be regarded as an important source of uncertain information and handled cautiously. The proposed method introduces a new method of addressing the uncertainty degree and fusion method of negation evidence simultaneously. The experimental results of experiments with artificial data and in fault diagnosis verify the rationality and effectiveness of the proposed methods.

It should be noted that there are limitations in the work. First, many new measures in Dempster–Shafer evidence theory framework are not taken into consideration in addressing the uncertain degree of negation evidence. Second, how to measure the uncertainty among dependent bodies of evidence is ignored in the proposed method. Third, other improved combination rules can be taken into consideration for evidence fusion. Last but not least, only two data sets are adopted to illustrate and verify the effectiveness of the proposed method. The method limitation is not clear for many other data sets and cases in artificial intelligence.

According to the aforementioned limitations, possible future research work is as follows. On the one hand, there is still no uncertainty measure that is accepted by all researchers in the evidence theory. A general uncertainty measure for negation evidence needs further study. On the other hand, although uncertainty measure-based weighted BPA has been widely used in multi-source information fusion, it cannot address the issue of dependent evidence fusion. How to address conflict and dependent evidence considering the uncertainty measure is a promising topic in multi-source information fusion.

## 5. Conclusions

In this paper, in Dempster–Shafer evidence theory framework, the belief entropy of negation evidence is proposed based on the belief entropy for the negation of BPAs. The measure for negation evidence is a new perspective on uncertain information modeling and measuring. Based on the belief entropy of negation evidence, a novel method considering BPA uncertainty degree in the negation information of multi-source information fusion is proposed. An experiment on artificial data and an application on fault diagnosis are designed to demonstrate the availability and effectiveness of the proposed measure and information fusion method. Both experimental results indicate that the belief entropy of negation evidence is effective in uncertainty measuring of negation evidence, and it contributes to an accurate evidence fusion result.

## Figures and Tables

**Table 1:** BPAs of multi-source information in the experiment with artificial data.

BPA	*m*(*A*)	*m*(*B*)	*m*(*C*)	*m*(*A,C*)
$m_{1}\left(\cdot \right)$	0.41	0.29	0.3	0
$m_{2}\left(\cdot \right)$	0	0.9	0.1	0
$m_{3}\left(\cdot \right)$	0.58	0.07	0	0.35
$m_{4}\left(\cdot \right)$	0.55	0.1	0	0.35
$m_{5}\left(\cdot \right)$	0.6	0.1	0	0.3

**Table 2:** Experimental results with different methods.

Methods	*m*(*A*)	*m*(*B*)	*m*(*C*)	*m*(*A,C*)
Deng et al’s method [46]	0.9820	0.0039	0.0107	0.0034
Zhang et al’s method [47]	0.9820	0.0033	0.0115	0.0032
Yuan et al’s method [48]	0.9886	0.0002	0.0072	0.0039
Xiao’s method [27]	0.9905	0.0002	0.0061	0.0043
Proposed method	0.9863	0.0013	0.0086	0.0038

**Table 3:** Data for fault diagnosis modeled as BPAs.

	Freq1				Freq2			Freq3		
	{F2}	{F3}	{F1,F2}	{F1,F2,F3}	{F2}	{F1,F2,F3}	{F1}	{F2}	{F1,F2}	{F1,F2,F3}
$m_{s_{1}}\left(\cdot \right)$	0.8176	0.0003	0.1553	0.0268	0.6229	0.3771	0.3666	0.4563	0.1185	0.0586
$m_{s_{2}}\left(\cdot \right)$	0.5658	0.0009	0.0646	0.3687	0.7660	0.2341	0.2793	0.4151	0.2652	0.0404
$m_{s_{3}}\left(\cdot \right)$	0.2403	0.0004	0.0141	0.7452	0.8598	0.1402	0.2897	0.4331	0.2470	0.0302

**Table 4:** Belief entropy of negation evidence of sensor reports under different frequencies.

Evidence	Freq1	Freq2	Freq3
$E_{n}\left(m_{s_{1}}\right)$	3.1726	2.7405	3.3109
$E_{n}\left(m_{s_{2}}\right)$	3.0142	2.9352	3.2634
$E_{n}\left(m_{s_{3}}\right)$	2.6191	2.9985	3.2789

**Table 5:** The weight of different sensor reports under different frequencies.

Evidence	Freq1	Freq2	Freq3
$w_{S_{1}}$	0.3603	0.3131	0.3360
$w_{S_{2}}$	0.3423	0.3398	0.3312
$w_{S_{3}}$	0.2974	0.3471	0.3328

**Table 6:** Modified mass function.

	Freq1				Freq2			Freq3		
	{F2}	{F3}	{F1,F2}	{F1,F2,F3}	{F2}	{F1,F2,F3}	{F1}	{F2}	{F1,F2}	{F1,F2,F3}
$m_{w}\left(\cdot \right)$	0.5597	0.0005	0.0823	0.3575	0.7538	0.2462	0.3121	0.4349	0.2098	0.0431

**Table 7:** Sensor data fusion results for fault diagnosis.

	Freq1				Freq2			Freq3		
	{F2}	{F3}	{F1,F2}	{F1,F2,F3}	{F2}	{F1,F2,F3}	{F1}	{F2}	{F1,F2}	{F1,F2,F3}
Fusion result	0.9146	0.0002	0.0394	0.0458	0.9851	0.0149	0.3353	0.6316	0.0329	0.0002

**Table 8:** Sensor data fusion results of different methods.

Method	Freq1				Freq2			Freq3		
	{F2}	{F3}	{F1,F2}	{F1,F2,F3}	{F2}	{F1,F2,F3}	{F1}	{F2}	{F1,F2}	{F1,F2,F3}
Jiang et al’s method [49]	0.8861	0.0002	0.0582	0.0555	0.9621	0.0371	0.3384	0.5904	0.0651	0.0061
Tang et al’s method [50]	0.8891	0.0003	0.0427	0.0679	0.9784	0.0216	0.3318	0.6332	0.0349	0.0001
Proposed method	0.9146	0.0002	0.0394	0.0458	0.9852	0.0149	0.3353	0.6316	0.0329	0.0002

## Data Availability

All data generated or analysed during this study are included in this published article.
